# Inequalities in Exposure to Nitrogen Dioxide in Parks and Playgrounds in Greater London

**DOI:** 10.3390/ijerph16173194

**Published:** 2019-09-01

**Authors:** Charlotte E. Sheridan, Charlotte J. Roscoe, John Gulliver, Laure de Preux, Daniela Fecht

**Affiliations:** 1 MRC Centre for Environment & Health, School of Public Health, Imperial College London, Norfolk Place, London W2 1PG, UK; 2 Centre for Environmental Health and Sustainability & School of Geography, Geology and the Environment, University of Leicester, University Road, Leicester LE1 7RH, UK; 3 Centre for Health Economics and Policy Innovation, Imperial College Business School, Imperial College London, Exhibition Road, London SW7 2AZ, UK

**Keywords:** nitrogen dioxide, children, exposure, play area, green space, London

## Abstract

Elevated levels of nitrogen dioxide (NO_2_) have been associated with adverse health outcomes in children, including reduced lung function and increased rates of asthma. Many parts of London continue to exceed the annual average NO_2_ concentration of 40 µg/m^3^ set by the EU directive. Using high-resolution maps of annual average NO_2_ for 2016 from the London Atmospheric Emissions Inventory and detailed maps of open spaces from Britain’s national mapping agency, Ordnance Survey, we estimated average NO_2_ concentrations for every open space in Greater London and analysed geospatial patterns comparing Inner verses Outer London and the 32 London Boroughs. Across Greater London, 24% of play spaces, 67% of private parks and 27% of public parks had average levels of NO_2_ that exceeded the EU limit for NO_2_. Rates of exceedance were higher in Inner London; open spaces in the City of London had the highest average NO_2_ values among all the London Boroughs. The closest play space for more than 250,000 children (14% of children) under 16 years old in Greater London had NO_2_ concentrations above the recommended levels. Of these children, 66% (~165,000 children) lived in the most deprived areas of London, as measured by the Index of Multiple Deprivations, where average NO_2_ concentrations in play spaces were on average 6 µg/m^3^ higher than for play spaces in the least deprived quintile. More action is needed to reduce NO_2_ in open spaces to safe levels through pollution reduction and mitigation efforts, as currently, open spaces in Greater London, including play spaces, parks and gardens, still have dangerously high levels of NO_2_, according to the most recent NO_2_ map.

## 1. Introduction

Exposure to nitrogen dioxide (NO_2_) has been associated with a wide range of negative health effects [1]. The damaging effects of NO_2_ exposure are particularly pronounced in children. Air pollution-related health effects in children include reduced respiratory function [2], asthma [3], obesity [4], and reduced cognition [5]. A study of school children in London found associations of NO_2_ exposure with long-term reductions in lung capacity (forced vital capacity [FVC]) [6]. In Australia, decreased lung capacity was measured even in the presence of relatively low levels of ambient NO_2_ [7] and, in Southern California, the magnitude of lung damage from NO_2_ exposure was roughly equivalent to maternal smoking [8]. 

The European Union (EU) mandates, in accordance with the World Health Organisation (WHO) guidelines, that ambient NO_2_ concentrations should not exceed an annual mean of 40 µg/m^3^ or an hourly mean of 200 µg/m^3^ more than 18 times per year [9,10]. Despite national and local policies, such as Air Quality Management Areas [11] and supporting tools for local authorities [12] to reduce air pollution, monitoring data from fixed sites continue to show illegal levels of NO_2_ in large parts of the UK, particularly in London. In 2016, 37 out of the 67 monitoring sites in London exceeded the EU annual limits and 23% of London residents lived in areas with illegal annual average levels of NO_2_ [13,14]. Pockets of the capital city, including Oxford Street, Brixton Road and Putney High Street, continue to record some of the highest annual mean values in Europe, belying overall improvements to air quality [13]. 

In response to continuing evidence on the health impacts of air pollution on children, a report by the Mayor of London’s office has examined NO_2_ levels in proximity to schools and found that over 400 primary schools in Greater London were exposed to NO_2_ levels in excess of the legal pollution limits [15]. Throughout the UK, over 2000 nurseries are within 150 m of a road that exceeds allowable levels of NO_2_ [16]. These studies have focused on outdoor NO_2_ concentrations to predict exposure for places where children spend large amounts of time indoors. Yet, a notable discrepancy exists between indoor and outdoor levels of NO_2_, with buildings providing a buffer from pollutants and mitigating against some of the outdoor exposure at indoor locations [17].

Outdoor spaces, such as parks and playgrounds, are frequently used for play and physical activity which requires increased air intake and circulation, facilitating inhalation of pollutants. While exercise is irrefutably linked to improved health, high levels of NO_2_ appear to temper those benefits [18,19]. Studies in children are sparse. However, Sinharay et al. found, in adults walking on Oxford Street, a highly polluted area relative to other parts of London, attenuated cardiopulmonary benefits typically observed with exercise [20]; while Strak et al. established, for cyclist commuters, an association of air pollution with airway inflammation [21].

London’s parks and gardens are renowned worldwide and often praised as London’s green lungs where residents can seek refuge from high levels of pollution, play and exercise. Two recently released datasets contradict this view of London’s green spaces. In this study, we integrate 2016 air quality data from the London Atmospheric Emissions Inventory with Ordnance Survey (OS) open space geographic data and the Office for National Statistics (ONS) census data to provide a detailed assessment of annual average NO_2_ concentrations across open spaces in Greater London with a focus on play areas and parks. We contrasted NO_2_ levels across different types of open spaces and quantify the spatial variation in average NO_2_ concentrations in London. We particularly highlighted air pollution exposure in children, linking census data to air quality data to estimate the number of children affected and highlighted socioeconomic inequalities across communities. 

## 2. Materials and Methods

To provide a detailed understanding of NO_2_ exposure across open spaces in Greater London, we used publicly available data on open spaces, annual average NO_2_ concentrations (µg/m^3^) and population characteristics.

### 2.1. Open Spaces Data

The Ordnance Survey (OS) ‘Open Greenspace’ data provides a reliable source of publicly accessible open spaces, verified by a community of users, with a high level of accuracy and detail. We supplemented the OS data with data on private parks from Greenspace Information for Greater London (GiGL). We extracted data for Greater London, defined as the 32 London Boroughs and the City of London. Open spaces extending beyond the study area were included if more than 25% of their area was contained within Greater London. 

**Figure 1:**
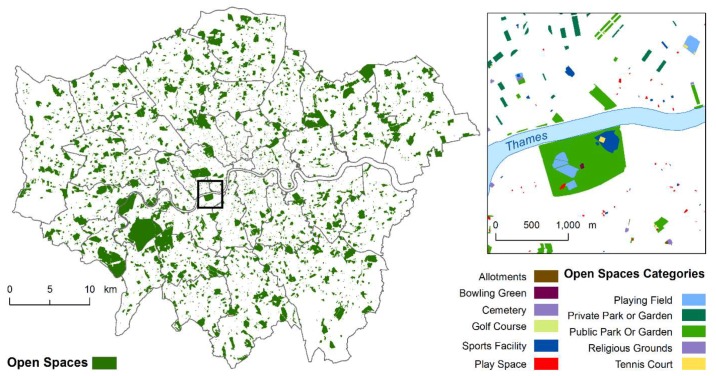
Open spaces in Greater London (within the extent of the 32 London Boroughs and the City of London) by category. [Contains OS Open Data^©^ Crown copyright and database right 2019; OS licenced data^©^ Crown copyright and database rights 2019 Ordnance Survey (100025252) and data provided by Greenspace Information for Greater London CIC, with permission to publish for illustrative purposes only, 2019].

Open spaces were classified as allotments, bowling greens, cemeteries, golf courses, other sports facilities, play spaces, playing fields, private parks, public parks or gardens, religious grounds, and tennis courts (Figure 1). Play spaces were defined as areas for private or public children’s play with “purpose-built equipment”, private parks were defined as parks and gardens that are “accessible only to certain people or where there is a financial charge for access” [22], and public parks or gardens (referred from here on as public parks) were defined as areas of land designated as such by a local authority with a distinct perimeter and free public access.

### 2.2. NO_2_ Concentrations

**Figure 2:**
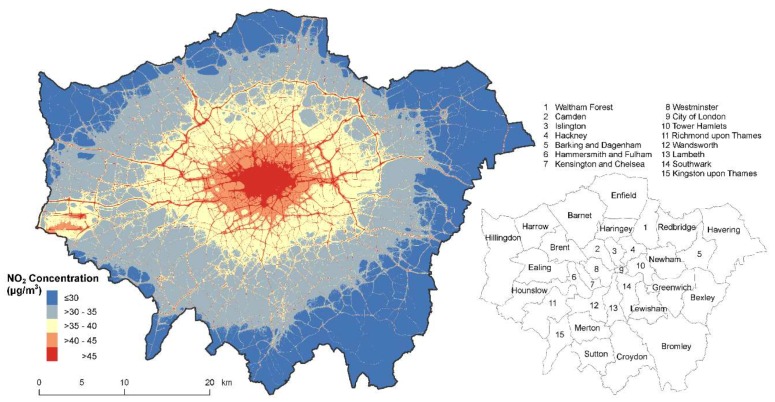
Annual average NO_2_ concentrations in Greater London from the London Atmospheric Emissions Inventory 2016, 20 m resolution and London Boroughs [Contains public sector information licensed under the Open Government Licence v2.0].

We obtained information on annual average NO_2_ concentrations from the London Atmospheric Emissions Inventory (LAEI) 2016 developed by King’s College London [23]. The LAEI is considered the primary authority for air pollution levels in London with annual average NO_2_ concentrations calculated as a 20 m × 20 m grid surface. The model uses 2016 emissions data from industrial, commercial, domestic, transport, and miscellaneous sources from a variety of datasets, including the Environment Agency’s Pollution Inventory and Local Authority records. These emissions are used in an atmospheric dispersion model to estimate ground level concentrations of NO_2_. Figure 2 shows the 2016 annual average NO_2_ levels from the LAEI with areas in red signifying NO_2_ levels above an annual average of 40 µg/m^3^. Policymakers at the Mayor’s Office and researchers worldwide use the LAEI model to represent air quality throughout the city.

To estimate NO_2_ concentrations in open spaces, we overlaid LAEI 2016 with the open space boundaries. NO_2_ concentrations from 20 m × 20 m data points (i.e., the centroid of grid squares) were averaged across each open space to estimate annual mean NO_2_ concentrations. If no data point was contained within the open space boundaries, we used the nearest data point to assign a NO_2_ concentration value. This was the case for 10% of open spaces, mostly play spaces, tennis courts and other sport facilities.

### 2.3. Socioeconomic Deprivation

We used the Index of Multiple Deprivation (IMD) 2015, a composite area-level measure of deprivation to estimate the socioeconomic status of the local area of play spaces. The IMD is used by local governments to quantify the socioeconomic status of neighbourhoods. The IMD is a weighted average of seven domains indicative of income, employment, education, health, crime, housing, and living environment deprivation. Each domain consists of several indicator variables (38 in total) which are combined using shrinkage estimation and factor analyses to derive domain scores [24]. The IMD for each lower layer super output area (LSOA, ~1700 residents in London) were obtained from the ONS. We categorised IMD scores into quintiles standardised for London to capture relative differences in deprivation across the city. To eliminate ambiguity over play spaces located across multiple LSOAs, play spaces were assigned an IMD quintile from the LSOA containing the location of the play space centroid. Socioeconomic status was only assigned to play spaces but not parks because parks often span many LSOAs in different socioeconomic quintiles which would make assignment to a specific socioeconomic quintile not feasible and increase uncertainty in results.

### 2.4. Child Residential Proximity to Play Spaces

To assess the number of children which potentially use a play space, we estimated the number of children under the age of 16 years in the local area using information from the 2011 census. We linked residential postcode centroids (average of 58 residents) to census output area (COA, average of 325 residents), the highest geographical level for which information on the number of under 16-year-olds is available. We used ONS 2011 postcode headcount data to estimate the number of children at each postcode centroid by multiplying the total population for a given postcode by the ratio of children under 16 years from the COA. In addition, IMD quintiles were assigned to each postcode based on the LSOA. All children within a postcode were then assigned to the nearest play space based on the distance from the postcode centroid to the play space.

### 2.5. Statistical Analysis

Our units of analysis were individual open spaces for which we compared average NO_2_ levels in 2016 and the percentage of open spaces with average NO_2_ levels in excess of the legal limit, 40 µg/m^3^, across different categories of open space. We explored spatial patterns by comparing metrics for each London Borough (see Figure 2), for Central versus Outer London and by deprivation bands (for play spaces only). We also assessed children’s exposure at their nearest play space and evaluated corresponding exposure inequalities across deprivation bands (quintiles) using independent-sample *t*-test.

## 3. Results

We analysed all 4470 open spaces in Greater London, including 3177 play spaces, 153 private parks and 1140 public parks. Play spaces and parks are prevalent throughout Greater London but differ in their usage and size. Play spaces are generally small, with 54% of play spaces encompassing an area of less than 500 m^2^.

Parks exhibited a wider range of areas with 45% private and public parks falling below 10,000 m^2^, although 16% of private and public parks span more than 100,000 m^2^. Some of London’s famous landmarks mirror this area distribution, with Tower Gardens and Parliament Square Gardens less than 10,000 m^2^ while Hyde Park and Regent’s Park easily surpass 100,000 m^2^. Large private parks include Kew Gardens and Buckingham Palace gardens. Gaseous pollutants such as NO_2_ have a steep distance-decay gradient away from traffic sources [25] which means that average pollution levels within the park are often a function of their size.

### 3.1. Average NO_2_ Concentrations in Open Spaces

Table 1 shows the substantial difference in average NO_2_ levels across different open space categories. For example, 24% of play spaces, 67% of private parks and 27% of public parks exceeded the annual average legal limit of 40 µg/m^3^ in 2016 (see Table 1). For most of these spaces (24%, 65% and 23%, respectively) the entire open space was above the legal limit. On the other hand, less than 5% of allotments, playing fields and golf courses exceeded on average the NO_2_ legal limit. Conker Tree play area, a play space located in the Royal Borough of Kensington and Chelsea and nestled between the on and off ramps of the A40 (one of the main arteries in London) had one of the highest NO_2_ concentration of any open space with an annual average of 64.3 µg/m^3^.

**Figure 3:**
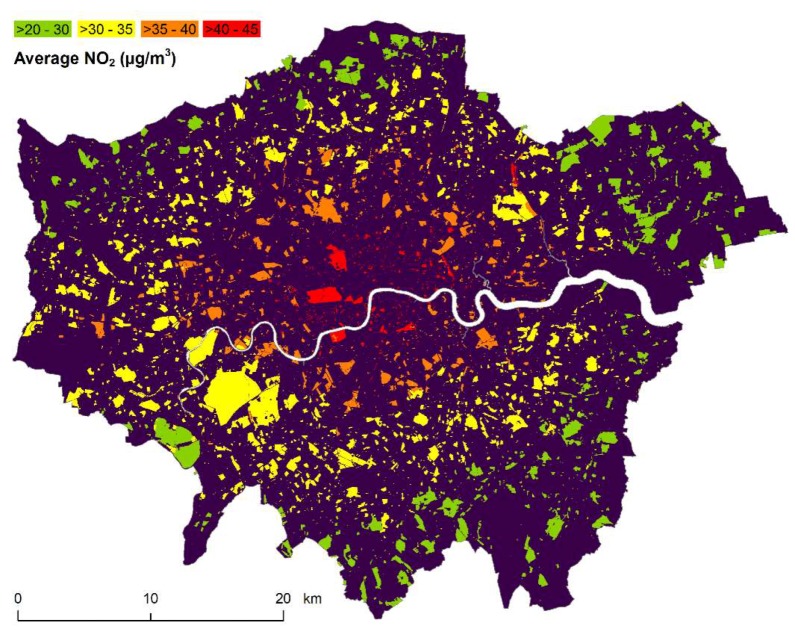
2016 annual average of NO_2_ concentrations across open spaces in Greater London, areas above the 40 µg/m^3^ legal limit shown in red [Contains OS Open Data^©^ Crown copyright and database right 2019; OS licenced data^©^ Crown copyright and database rights 2019 Ordnance Survey (100025252) and data provided by Greenspace Information for Greater London CIC, with permission to publish for illustrative purposes only, 2019; public sector information licensed under the Open Government Licence v2.0].

Five of the top 10 most polluted parks can be found in Westminster. Victoria Embankment Gardens, located along the River Thames and near the House of Parliament had the highest annual average NO_2_ concentration (59.7 µg/m^3^) across all public parks, closely followed by Parliament Square Garden with 58.3 µg/m^3^. In general, Central London has a disproportionately large number of open spaces above the 40 µg/m^3^ legal limit (see Figure 3). In Central London, 44% of play spaces exceeded this threshold compared to 2% in Outer London. Likewise, 83% of private parks and 55% of public parks in Central London surpassed the limit compared to 0% and 4%, respectively, in Outer London. Figure 3 highlights this spatial clustering of open spaces above the legal limit in Central London. Echoing this pattern, all London Boroughs with the highest number of open spaces were average NO_2_ levels are above the legal limit are in Central London (Table 2). In the City of London, all play spaces and private and public parks had an annual average concentration of NO_2_ exceeding the legal limit, followed by Westminster with 96%, 100% and 98% of play spaces, private and public parks, respectively above the limit.

### 3.2. Inequalities in Children’s Exposure at Play Spaces

**Figure 4:**
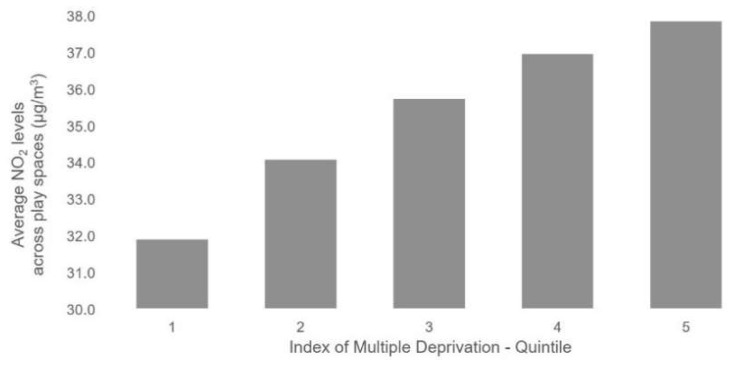
Average NO_2_ concentrations across play spaces by deprivation quintiles (i.e., 5ths), where 1 is least deprived and 5 is most deprived.

Our analysis of play space revealed a strong positive relationship of increasing average NO_2_ levels with increasing deprivation. Independent-sample *t*-tests indicated significant differences in NO_2_ concentrations between deprivation quintiles (*p* < 0.01). The average NO_2_ level for play spaces in the most deprived quintile was 6 µg/m^3^ higher than the average NO_2_ level for those in the lowest quintile (see Figure 4). Play spaces with concentrations above the legal limit were, however, not evenly distributed across deprivation quintiles, with 39% of these play spaces located in the most deprived areas compared to 9% of these play spaces in the least deprived areas.

**Figure 5:**
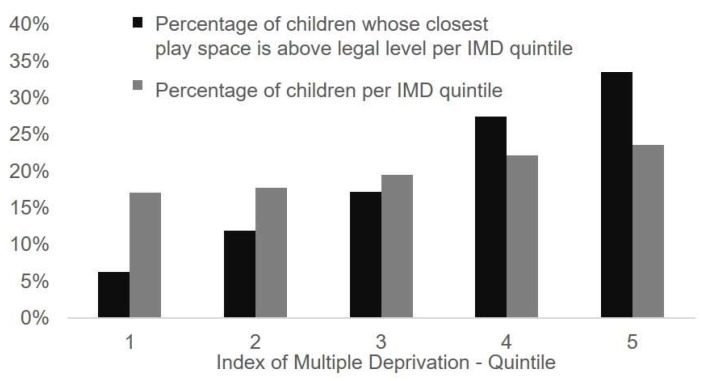
Percent of children in Greater London per Index of Multiple Deprivation quintile (grey) and percentage of children per Index of Multiple Deprivation quintile whose nearest play space exceeds the limit for NO_2_ (black).

For more than 250,000 children (14%) under 16 years old in Greater London, their closest play space exceeds the legal limit for NO_2_. Of these children, 66% (~165,000 children) live in areas in the 4th or 5th most deprived quintile (see Figure 5). The difference in proportion between children in the most compared to the least deprived areas whose nearest play space exceeds the legal limit is 19%, highlighting the unequal distribution of NO_2_ levels across deprivation bands. 

## 4. Discussion

Our study is the first to evaluate the levels of NO_2_ concentrations in open spaces focusing on parks and play spaces in London and their relationship with deprivation. This research adds to the body of studies highlighting the health effects of air pollution in children and differences in NO_2_ exposure across socioeconomic status.

### 4.1. Key Results

Using modelled NO_2_ concentrations for 2016, 750 play spaces, 99 private parks and 264 public parks exceeded the legal limit for annual average NO_2_ of 40 µg/m^3^. Private parks had the highest average NO_2_ levels across all open spaces in London and were more likely to exceed the legal limit of 40 µg/m^3^ compared to other types of open space. Given the prevalence of private parks in wealthy neighbourhoods, the poor air quality in private parks presents a seemingly contradictory narrative to the association between deprivation and air quality levels. Indeed, 63% of private parks are located in the two least deprived quintiles of deprivation (quintiles 1 and 2), despite an average NO_2_ level of 41 µg/m^3^. The high levels of NO_2_ concentrations in private parks, however, can best be attributed to mostly being found in Inner London (80%) which has considerably higher levels of NO_2_. Private parks do not accurately portray city-wide pollution in open spaces because they represent a small subset of open spaces (1.5%) and are largely located in two boroughs: Westminster and Kensington and Chelsea.

Four of the worst polluted 10 parks or gardens—Parliament Square Garden, New Palace Yard, Victoria Tower Gardens, and the Speaker’s Green—are in close proximity to the Palace of Westminster. Average annual NO_2_ levels at these relatively small spaces are particularly high due to their location amongst busy streets, and levels during rush hour can be presumed to far exceed this annual average.

The 750 play spaces exceeding the legal NO_2_ limit serve up to a quarter of a million children under 16 years old in London. We used the most recent data (2016) to represent NO_2_ concentrations in open spaces. Levels of NO_2_ may have generally fallen since 2016, following the slow decreasing trends over the last decade, but there is currently no mapping available for 2017 and 2018. Potential decreases in NO_2_ levels since 2016 and into the future may not change the health outcome for thousands of children who frequented play spaces prior to air quality improvements. The evidence is inconclusive as to whether health effects could be fully or partially reversed. In the California Children’s Health Study, researchers observed improved lung growth trajectories after decreases in air pollution. However, further studies would be necessary to extrapolate findings from California, where fine particulate matter from petrol vehicles is of primary concern, to European cities such as London, which has exceptionally high local levels of NO_2_ primarily from diesel traffic. 

The burden of NO_2_ pollution is not shared equally among London residents. Our analysis revealed the extent of the inequality across local areas with play spaces in the most deprived quintile being six times more likely than those in the least deprived to exceed the legal limit for NO_2_. This finding mirrors a previous analysis by Fecht et al. which highlighted the significant difference in NO_2_ exposure between the most and least deprived quintiles in London [26]. Although the placement of more play spaces within deprived areas appears to be positive action, those spaces have worse air quality, which may lead to greater health impacts, increasing health inequalities between the most and least deprived quintiles. 

Spatial variations in NO_2_ are highly influenced by the steep distance-decay gradient of NO_2_, around local sources. According to the Department of Environment, Food & Rural Affairs (Defra) guidance, spikes in NO_2_ levels that occur near intersections and other sources of roadway emissions return to background levels within 50 m [25]. In the case of London, the most significant source of NO_2_ is road vehicle emissions, making placement of an open space relative to the nearest roadway an important consideration. To protect children from the negative health impacts related to air pollution, urban planning should consider the placement of play spaces in relation to traffic sources. Improving the design of urban streets to provide better ventilation and strategic use of vegetation to maximise the potential for absorption of air pollutants [27], coupled with measures to generally reduce traffic volumes, can promote cleaner air. Many play spaces were set within larger parks, in particular in the least deprived areas, which may have provided a buffer from neighbouring road traffic and explained some of the discrepancy in NO_2_ levels across deprivation quintiles.

### 4.2. Policy Implications

As the only comprehensive analysis of NO_2_ levels at open spaces in London, we have shown that not all public parks and play spaces suffer equally from high levels of air pollution. Average NO_2_ concentration values for open spaces, even in Inner London, range from 23 µg/m^3^ to 66 µg/m^3^. Our analysis has highlighted the parks and play spaces with the highest average NO_2_ levels and those with their total surface area above the limit for NO_2_—those parks would benefit most from specifically targeted air quality improvement measure. Building on ongoing efforts to reduce air quality levels in London, such as the Ultra-Low Emissions Zone (ULEZ), additional localised measure could be put in place specifically to target improving air quality in the most polluted parks and play spaces. Such solutions could include “pedestrianisation” of traditional streets or car-free days, as many other inner cities have done, to limit pollution [28]. Play spaces immediately adjacent to busy intersections could be partially protected with low porosity vegetation or green walls which mitigate some exposures [29] but do not reduce overall air pollution.

Much of the Mayor of London’s Local Air Quality Management Framework (LLAQM) devolves responsibilities to individual London Boroughs for air quality monitoring and improvement plans. Boroughs with parks or play spaces that were most affected by NO_2_ pollution can use this information to inform air quality management. Until effective policies are implemented, children and adults may consider avoiding open spaces with harmfully high levels of NO_2_, to reduce prolonged exposure.

### 4.3. Strengths and Limitations

All analyses relied on the accuracy of the LAEI modelled NO_2_ data and interpolation therein from London Air Quality Network (LAQN) monitoring averages. The LAEI surface is one of the most accurate air quality models globally and LAQN monitoring is the most comprehensive available for Greater London effectively reducing study limitations. Calculations on the impact of NO_2_ pollution on children at play spaces were limited by uncertainty surrounding the behaviour of London residents in selecting open spaces to visit. We assumed that children’s exposure occurs at their local play space, which may not be true in all cases. Despite this uncertainty, our figures are revealing of the overall magnitude of NO_2_ exposure at play spaces and a reasonable estimate of child exposure. Due to data limitations, our study examined only annual average values for NO_2_ although play spaces tend to be in use during the day when pollution-generating road traffic is heavier and consequently, the real exposures at play spaces may be higher than the annual average suggest. Moreover, both outdoor play and air pollution levels vary seasonally and this seasonal variability should be further assessed if such data becomes available. 

## 5. Conclusions

We integrated newly released air quality data from the Mayor of London for 2016 with Ordinance Survey open space data to highlight the scale of the air pollution problem in open spaces in Greater London. We found that 24% of play spaces, 65% of private parks and 23% of public parks were above the legal limit of 40 µg/m^3^ annual average NO_2_ concentrations. These spaces often used for physical activity and a high level of pollutant intake is expected due to higher breathing rate, making this a potentially large public health issue.

## Figures and Tables

**Table 1:** Average nitrogen dioxide (NO_2_) concentrations in open spaces by category, including the percentage of open spaces above the legal limit of 40 µg/m^3^.

Open Space Category	*N*	Average NO_2_ Concentration (µg/m^3^)			Percentage of Open Spaces with NO_2_ > 40 µg/m^3^		
		Mean	Min	Max	Average NO_2_ > 40 µg/m^3^	100% Area >40 µg/m^3^	>1% Area >40 µg/m^3^
Allotment	769	33.0	23.9	45.9	4%	4%	9%
Bowling Green	301	32.8	24.4	44.9	2%	2%	4%
Cemetery	209	34.4	24.2	56.3	12%	9%	33%
Golf Course	131	30.2	22.7	46.7	2%	2%	24%
Sports Facility	1524	35.3	23.5	66.0	18%	17%	24%
Play Space	3177	36.3	23.3	64.3	24%	24%	25%
Playing Field	738	32.1	23.3	46.2	3%	2%	14%
Private Park or Garden	153	40.5	27.6	57.7	67%	65%	78%
Public Park or Garden	140	36.4	24.3	59.8	27%	23%	46%
Religious Ground	802	34.6	23.6	53.2	13%	10%	21%
Tennis Court	1000	33.4	23.4	52.5	6%	6%	7%

**Table 2:** Annual average NO_2_ concentrations in play spaces, private parks and gardens and public parks and gardens by London Boroughs (ranked by average NO_2_ levels in play spaces), including the percentage of spaces above the legal limit of 40 µg/m^3^.

Borough	Play Spaces		Private Parks and Gardens		Public Parks and Gardens	
	Average NO_2_ (µg/m^3^)	% Spaces > 40 µg/m^3^	Average NO_2_ (µg/m^3^)	% Spaces > 40 µg/m^3^	Average NO_2_ (µg/m^3^)	% Spaces > 40 µg/m^3^
City of London *	45.3 ^†^	100	53.7 ^†^	100	50.1 ^†^	100
Westminster *	44.0 ^†^	96	46.4 ^†^	100	47.3 ^†^	98
Camden *	42.1 ^†^	72	42.4 ^†^	50	43.7 ^†^	80
Kensington and Chelsea *	41.5 ^†^	72	42.1 ^†^	93	42.6 ^†^	87
Islington *	41.3 ^†^	66	-	-	42.6 ^†^	76
Southwark *	41.1 ^†^	64	-	-	41.4 ^†^	57
Tower Hamlets *	41.1 ^†^	69	41.8 ^†^	71	42.5 ^†^	79
Lambeth *	40.0 ^†^	45	42.8 ^†^	67	42.2 ^†^	64
Hackney *	39.5	35	-	-	40.4 ^†^	56
Hammersmith and Fulham *	39.5	26	-	-	40.0	32
Wandsworth *	37.3	16	37.2	0	38.9	13
Newham	37.0	5	36.9	0	36.3	0
Brent	36.8	14	32.3	0	35.5	0
Haringey	36.3	3	-	-	37.5	15
Lewisham *	35.6	5	36.5	0	35.8	5
Ealing	34.9	3	35.5	0	36.7	17
Greenwich *	34.8	3	-	-	34.0	3
Waltham Forest	34.8	4	34.2	0	36.4	15
Hounslow	34.3	4	33.6	0	35.2	7
Barnet	34.0	4	-	-	33.6	8
Merton	33.1	0	33.0	0	34.1	7
Barking and Dagenham	33.0	0	-	-	31.5	0
Richmond upon Thames	32.7	1	31.9	0	33.5	0
Redbridge	32.7	5	-	-	33.9	13
Enfield	32.4	0	28.6	0	33.1	2
Kingston upon Thames	31.9	0	31.9	0	32.8	0
Hillington	31.2	0	33.2	0	31.2	0
Croydon	30.9	0	-	-	30.4	0
Sutton	30.5	0	-	-	31.1	0
Harrow	30.5	0	-	-	31.0	0
Bexley	29.8	0	-	-	30.1	0
Bromley	28.6	0	29.9	0	29.5	2
Havering	27.7	0	-	-	27.0	0

* Inner London Boroughs and City of London, ^†^ above the legal limit.
